# A review of lead times and determinant factors in public optometry services in South Africa

**DOI:** 10.4102/hsag.v31i0.3208

**Published:** 2026-08-06

**Authors:** Mapula P. Rapao, Pheagane M.W. Nkoana

**Affiliations:** 1 Department of Optometry, Faculty of Health Sciences, University of Limpopo, South Africa

**Keywords:** lead time, optometrists, optometry services, public hospital, waiting time

## Abstract

**Background:**

Lead time is the total time spent by a patient in a facility from their arrival until the end of the last service they receive. Increased waiting times, which are idle times, in between service points within a service system significantly lengthen the lead times.

**Aim:**

To review lead times and the determinant factors in public optometry services in South Africa.

**Methods:**

Literature was reviewed from Google Scholar, PubMed and ScienceDirect databases using key terms such as ‘lead time’, ‘waiting times’, ‘optometry services’ and ‘public hospitals’. To obtain more data about optometry services and wait times in public hospitals, seven websites were also included.

**Results:**

It was determined that 38 academic papers were pertinent to the review. Fifteen were optometry and eye care, six on lead time,six on triage, three queuing theory, one on patient flow, three on lean theory and two on frame work. Human resource limitations, insufficient equipment, unequal optometrist distribution and ineffective referral and patient flow mechanisms are among the main issues influencing lead times.

**Conclusion:**

To address the prolonged lead times for service in optometry, the report emphasises the urgent need for systemic changes in rural South African public hospitals. To ensure patients receive prompt and efficient eye care. It is imperative to increase the availability of qualified staff, upgrade infrastructure and expedite procurement procedures.

**Contribution:**

This study not only sheds light on existing debates on lead times and the system-level factors such as human resources, infrastructure and patient flow inefficiencies that influence them in South African public optometry services, but it also highlights important knowledge gaps and offers guidance for further studies and improvements in the healthcare system.

## Introduction

Availability, accessibility and affordability of eye care services are either poor or lacking in rural communities worldwide (Chen et al. 2025), mainly because of migration of eye care providers to urban areas for better economic opportunities, resulting in, among other things, a shortage of optometrists in rural areas (Mashige, Oduntan & Hansraj 2015). This phenomenon has been reported in numerous countries, including the United States (US) (Solomon et al. 2022), Australia (Chen et al. 2025) and South Africa (Zulu & Van Staden 2023). This trend is particularly evident in South Africa because a significant portion of the population lives in rural regions (Masemola, Baloyi & Xulu-Kasaba 2025). The availability, accessibility and affordability of optometric eye care services were identified as key factors influencing the utilisation of eye care services in rural areas. In South Africa, optometry services are mainly provided by the private sector, and consequently, many people, especially those who live in remote areas, because of the distances to the available facilities and because they cannot afford the service (Nkoana 2024). In certain instances, the services in these facilities are limited, hence reducing availability (Blanford et al. 2012). The need for eye care services in South Africa is growing (Masemola et al. 2025). The public health sector, which is the primary provider of care, is unable to cope with the demand because of the lack of funding, resulting in severe shortages in providing basic equipment, consumables and optical devices and qualified staff (Nkoana, Mashige & Moodley 2024). Masemola et al. (2025) emphasise how crucial operational effectiveness is to achieving the best possible patient results in speciality services like optometry. Operational effectiveness results in increased waiting time (WT) and consequently longer lead time (Palvannan & Teow 2012). Waiting time may be described as the idle time between activities or when the activities of a case are not being processed (Ali, Milani & Dumas 2025). In healthcare, this may imply the time the patient takes waiting for a doctor, nurse or pharmacist to receive an examination or medication (Department of Health 2023). Biya et al. (2022) describe lead time, also known as cycle time, as the total time a patient takes within the care pathway from when they enter a healthcare facility to completion of the service. According to Biya et al. (2022), lead times for scheduling an appointment or obtaining treatments on the day of the appointment vary across nations and even within the same nation among medical facilities. These variations may be affected by patients’ educational attainment, the time they arrive at the hospital (Biya et al. 2022) and the availability of workspace, equipment, medical supplies (e.g. optical devices) and medical staff (Woldeyohanins et al. 2025). These concepts originate from literature in manufacturing and operations management. Hence, WT is a component of the lead time. Some healthcare literature such as Pozzan et al. (2025) suggest overlap in using lead and wait times, as they are both used to measure timeliness, though they measure different forms of delays. Despite their frequent interchangeability, the phrases ‘waiting time’ and ‘lead time’ have different meanings in this study. Waiting time refers to the duration, in minutes or hours, between the moment a patient is added to a waiting list and the time they receive care or services (Department of Health 2023). Lead time, by contrast, is the duration of the entire process, including WTs at each service-delivery stage (Biya et al. 2022). Lack of human resources for eye care (HReH) contributes significantly to longer WTs to secure an appointment to receive public eye care services and longer queues when patients visit eye care facilities (Nkoana et al. 2024). Longer lead times, however, affect the satisfaction rate of users of the service and negatively limit the efforts of reducing visual impairment and blindness (Zulu & Van Staden 2023). Masemola et al. (2025) reported that some hospitals in rural South Africa, specifically in the Limpopo province, have few or no ophthalmic nurses and depend only on optometrists for eye care. Consequently, patients who rely on public services often experience longer lead times and extended WT for care, as facilities struggle to meet demand with limited personnel, infrastructure and resources, delaying access to essential optometry services and contributing to inequities in eye health outcomes. Though the incorporation of eye care in the public health system keeps on improving, there are still challenges (Zulu & Van Staden 2023). Longer waiting and lead times may result in reduced use of the service. Patients with sight-threatening conditions such as glaucoma may lose their sight while waiting or because of withdrawal from service. These patients will then experience poor quality of life, some being unable to work (Nkoana 2024). This review, therefore, outlines the importance of lead times in eye care, specifically within public optometry service in South Africa; examines queuing theory as a theoretical framework for lead times; explores the factors contributing to prolonged waiting and lead times for obtaining appointments and receiving care during consultations and reviews the South African legal framework on WTs.

## Research methods and design

### Scoping review

In assessing lead times and the determinant factors of WTs in public optometry services in South Africa and around the world, a scoping literature review design was used. The study adapted a scoping review approach to allow mapping of existing evidence, documentation of research gaps and a combination of various study designs relevant to health service delivery research. The Arksey and O’Malley outline, which consists of determining the research topic, looking for pertinent studies, choosing studies, charting the data and summarising findings, is frequently used to guide scoping reviews.

### Search strategy

Google Scholar, PubMed and ScienceDirect were used to search the literature. Additional grey literature was discovered through relevant organisational websites and policy documents, which strengthen peer-reviewed findings.

The following keywords and Medical Subject Headings were combined in the search strategy: ‘lead time’, ‘waiting time’, ‘optometry services’, ‘eye care services’, ‘public hospitals’, ‘patient flow’, ‘triage’ and ‘queuing theory’. Boolean operators (AND, OR) were employed to refine search results. In order to find more pertinent research, the reference lists of a few chosen papers were also carefully examined.

### Eligibility criteria

#### Inclusion criteria

Research was incorporated if they:

- Reported on waiting times, lead times or patient flow in optometry, ophthalmology or public healthcare settings.
- Were conducted in public hospitals, public clinics or government-funded health facilities.
- Were peer-reviewed journal articles, systematic reviews, policy reports or authoritative web-based sources.
- Were released in English.

#### Exclusion criteria

Research was not included if it:

- Totally focused on private healthcare facilities.
- Did not report waiting time or service delivery outcomes.
- Were abstracts from conferences, editorials or commentary that lacked comprehensive methodological information.

#### Study selection procedure

A systematic screening procedure was used to choose the study participants:

- Title screening to eliminate records that are not relevant.
- Abstract selection to determine the importance of lead times and optometry services.
- Full-text screening to confirm eligibility based on inclusion criteria.

This procedure was carried out to guarantee openness and reproducibility while reducing selection bias.

#### Data extraction

Data were extracted using a standardised data extraction template. The following variables were recorded:

- The author and the publishing year.
- Healthcare and the country.
- Study design and population characteristics.
- Reported lead times or waiting times.
- Factors influencing waiting times.
- Recommendations for reducing lead times.

### Data analysis and synthesis

Results were synthesised using a qualitative thematic analysis approach. The themes that emerged were divided into three categories: operational, systemic and resource-related elements that impact lead times. Because of the diverse study designs, results and measuring techniques that hindered quantitative meta-analysis, the narrative synthesis approach was chosen.

### Quality appraisal

The Joanna Briggs Institute (JBI) checklist and other well-known critical assessment instruments were used to evaluate the methodological quality of the included studies. To increase the validity of the results, this evaluation looked at study design, methodological rigour and bias risk.

### Ethical considerations

This article followed all ethical standards for research and does not contain any studies involving human participants performed by the author.

## Results

**FIGURE 1:**
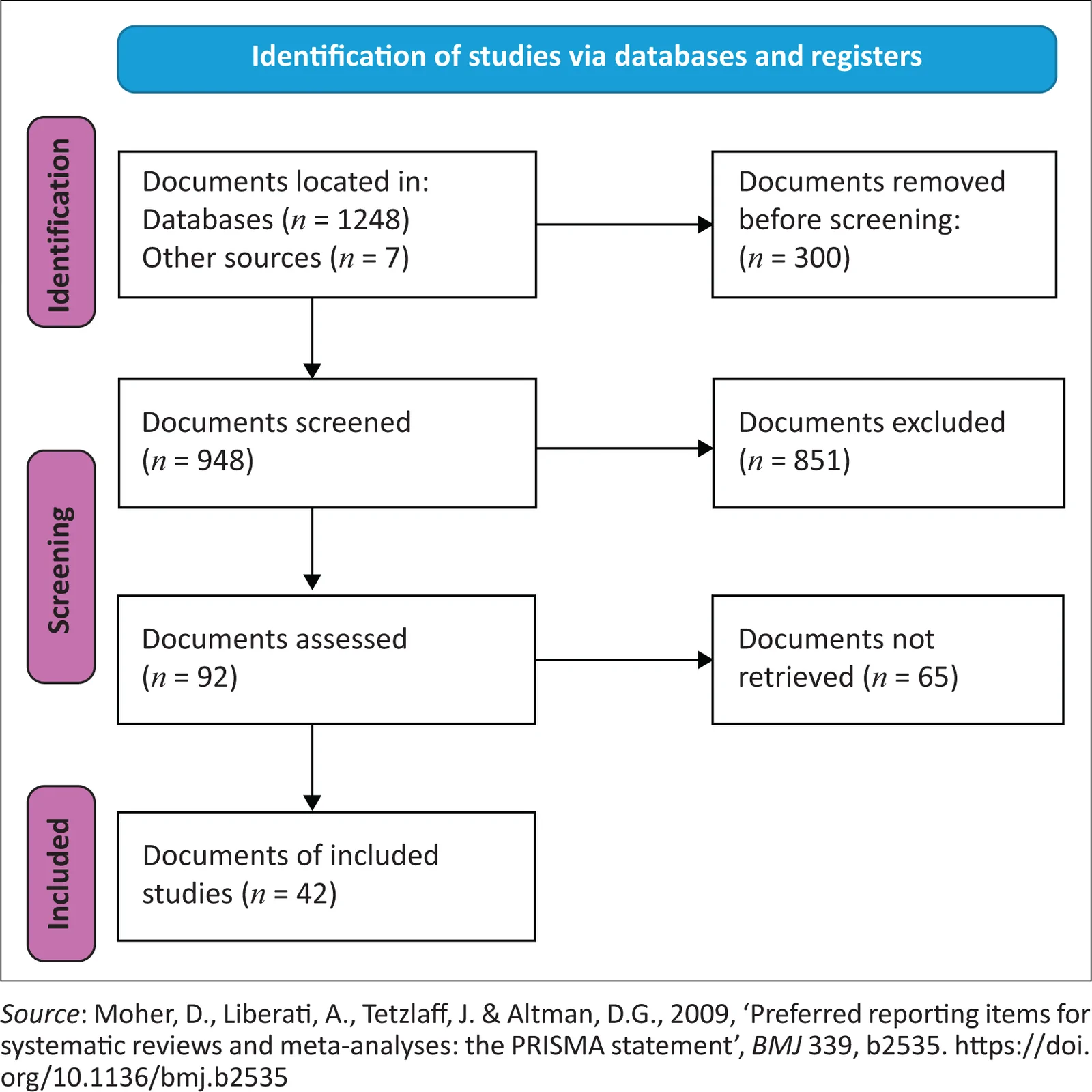
Preferred reporting items for systematic reviews and meta-analyses diagram.

As illustrated in Figure 1, database search yielded 1248 records, after title or abstract screening and duplicate removal, 312 advanced and then 92 for full-text review. Post-screening (excluding 65 irrelevant or private or shallow studies), 38 peer-reviewed articles qualified for synthesis, supplemented by four grey literature or policy sources on South African public optometry.

### The queuing theory

**FIGURE 2:**
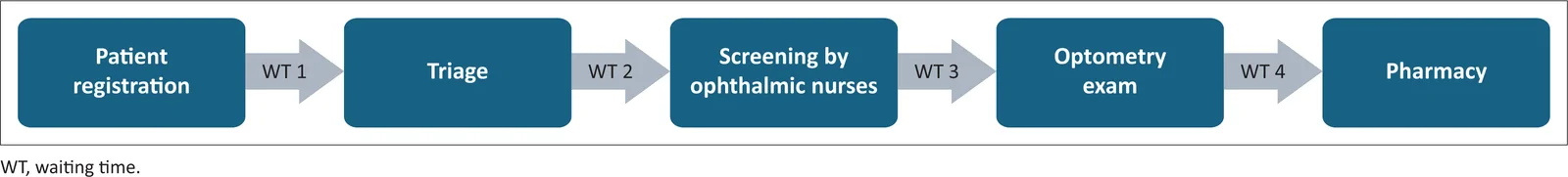
A simulated optometry service system.

The concept of waiting and lead times can be best understood through the queuing theory, which is a well-known method in operations research, offering a mathematical model for wait times analysis and optimisation in medical, banking and automotive industries and environments (Ameh, Sabo & Oyefabi 2013). It provides insightful tools for comprehending and maximising patient flow by portraying patients as ‘customers’ and the optometric service process as a ‘service system’. The service system in the context of optometry service, as shown in Figure 2, includes service steps from the registration of a patient, the triage, optometry examination and a visit to the pharmacy to receive medication. The time taken to open and receive the medical file, the time taken for triage, the time to receive an optometry and ophthalmic nurses’ service and the time to receive medication, plus all the waiting times (WT1, WT2, WT3, WT4) in between the service points, will constitute lead time. In cases of hospitals with ophthalmic nurses, patients do not have direct access to optometrists but are rather referred. Between these service points, patients experience WTs. The time patients spend waiting between service points, the WTs, lengthens the total time patients spend within the system, the lead times.

Queuing theory helps one to forecast WTs depending on elements such as patient arrival rates, healthcare provider service times and resource availability (Peter & Sivasamy 2021). Management of patient expectations and effective resource allocation depends on this predictive capacity (Peter & Sivasamy 2021). Furthermore, queuing theory can direct the creation of plans to lower delays using staffing level adjustments depending on patient demand or optimal appointment scheduling to minimise overlaps and gaps (Peter & Sivasamy 2021).

### Lean management method

In addition to the queuing theory, the lean management method is a useful framework as it emphasises spotting and cutting waste in medical procedures, including pointless WTs. This method stresses process optimisation, effective resource use and ongoing improvement, all of which can be utilised to increase the efficiency of optometric services and reduce lead times (Robinson et al. 2016). Lean management promotes the use of tools like value stream mapping to clearly show all phases involved in providing healthcare services, therefore enabling the identification and elimination of non-value-added activities, like insufficient patient scheduling and variability in patient arrival times, causing delays (Lawal et al. 2014). Lean management can be applied in this context by reducing WTs to consequently shorten lead times. An analysis of patterns of peak times or days where there is increased flow of patients to specific service points can be made and additional staff members scheduled based on need as depicted from such an analysis. Examples of lean management in cases where there are limitations of staff capacity may include rescheduling nurses from other units to triage for peak times to increase capacity during peak periods, like in the morning, when many patients visit these units before they are directed to other specific service points such as optometry, physiotherapy and dentistry.

### Lead times in healthcare and optometry services

Waiting times are rather critical to patient outcomes because waiting for an appointment may result in poor visual outcomes or partial or complete vision loss for conditions such as keratoconus, cataract and glaucoma (Masemola et al. 2025). Patients accessing optometry services in public hospitals often express significant dissatisfaction with long WTs, which negatively affect their overall experience and trust in the healthcare system. According to studies in African contexts such as South Africa and Kenya (Timothy et al. 2023; Zulu & Van Staden 2023), patients view lengthy WTs as a significant obstacle to receiving appropriate eye care, which can occasionally result in treatment delays and worsening of eye problems. These delays are caused in part by the large patient loads, scarce resources and ineffective clinic workflows in public facilities (Buthelezi & Van Staden 2020; Masemola et al. 2025). Studies show that a lack of transparency and inadequate communication regarding anticipated WTs increase patient annoyance (Ditibane 2024; Ritshidze.org.za 2023). According to Hansraj et al. (2024), patients report that WTs reduce their interest in consulting, and they are less likely to show up for follow-up appointments, which compromises continuity of care. Application concepts of lean management through the provision of information on the service and effective scheduling may reduce WTs and increase patient satisfaction with public optometry services. Healthcare financing, which is limited and unable to support the operations in South African public healthcare facilities, results in longer lead or WTs (Masemola et al. 2025). Hospital funding can reduce WTs by enabling hospitals to enhance their service capacity through the hiring of additional staff, the purchase of more advanced equipment and the improvement of infrastructure (Brindley, Lomas & Siciliani 2023; Masemola et al. 2025; Nkoana et al. 2024). Patients therefore wait for longer times to get appointments as resources are limited to carry the burden of need for healthcare. Even on the day of consultation, they experience longer lead times from the time they enter the facility to when they complete their consultation (Brindley, Lomas & Siciliani 2023). Participants observed that while those who received prompt care reported favourable experiences, patient satisfaction was significantly impacted by delays in cataract procedures and extended WTs for spectacles (Masemola et al. 2025). To draw parity for this paper, WTs will constitute the time a patient ‘spends waiting between the arrival at a service point and the time they receive services’ at that service point (Department of Health 2023).

A patient consulting at an eye clinic of a healthcare facility undergoes the registration process to acquire a medical record and sort out financial requirements, undergoes triage procedures to determine the presence and severity of systemic and underlying conditions such as diabetes and hypertension critical to visual outcomes and undergoes oculo-visual screening at the ophthalmic nurses’ station, if available, before they are referred to an optometrist for a full eye examination (Mbwogge et al. 2022). There are subsequent supplementary processes such as consultation services other than eye care and acquiring prescribed or recommended medication at the pharmacy section.

### Factors contributing to extended lead times

#### Limited human resources

The number of registered optometrists (4204) in South Africa is higher than the number of ophthalmologists (580) (Hansraj et al. 2024). An estimated 6.7% of optometrists work in the public sector, even though the majority (93.3%) work in the private sector. Lead times for consultations and operations are prolonged because of this shortage, increasing patient loads for the available optometrists (Hansraj et al. 2024). The lack of specialist personnel, especially ophthalmic nurses, dispensing opticians and ophthalmologists, is also a persistent issue. Generally, the shortage of HReH affects the standard of care, particularly for more complicated eye health issues, where patients must wait a lot longer to receive either basic or specialised care (Masemola et al. 2025).

#### Patient registration systems, triage procedures and the availability of supplementary services

Triage protocols, patient registration systems and the availability of additional services are all essential to providing high-quality healthcare. In a country riddled with significant non-communicable diseases, triage provides an appropriate platform to determine the priority patients based on their medical needs (Peter & Sivasamy 2021). While this is critical, the triage stage delays have the potential to seriously lengthen patient wait times and lower the standard of care (Peta et al. 2023). Furthermore, patient registration guarantees that the required data crucial for starting precise diagnostic and treatment regimens are gathered in any healthcare setting. How well these processes function has a direct impact on patient outcomes and the overall functioning of healthcare facilities.

#### Infrastructure constraints

The lack of or poor availability of resources and infrastructure exacerbates lead times. According to Zulu and Van Staden (2023), optometrists in South Africa, especially those in KwaZulu-Natal province, usually work without the required equipment and infrastructure, which affects care and increases WTs. This has also been reported in many hospitals in Limpopo province, where problems such as outdated technology, a lack of infrastructure and inadequate equipment were identified (Masemola et al. 2025). For patients who need surgery or spectacles, these shortcomings frequently lead to lengthy WTs; some patients may have to wait up to 5 years for care. Nkoana et al. (2024), on the other hand, reported that most hospitals did not have sufficient working space, lacked the equipment and materials needed for patient examination and could not provide optical devices. The availability of HReH, equipment and workspace or lack thereof, are critical contributing factors to increase or decrease in lead and WTs.

#### Referral system inefficiencies

To guarantee that patients receive the right care, an effective referral system is essential. Based on the primary health care and district health models adopted by the South African public health system, patients get referred by healthcare providers from clinics to hospitals or from one hospital to the next at district, regional and tertiary levels to access specialised services. Unlike the urbanised provinces such as Gauteng and the Western Cape, other provinces have fewer hospitals, especially tertiary and academic hospitals. Examples can be drawn from KwaZulu-Natal province with a population of about 12 423 907 the second largest in SA and Limpopo Province with 6 572 720 people but have three and one tertiary hospitals respectively (Census 2022, Ritshidze.org.za 2023). Delays in the referral procedures and the length of time patients must wait for additional medical interventions were commonly cited by participants in the study by Masemola et al. (2025) as obstacles to providing quality care.

#### Supply chain issues

Delays in the procurement and delivery of essential optical supplies, such as prescription spectacles, exacerbate lead times. Patients have reported waiting for extended periods to receive their eyewear, with some experiencing worsened vision because of the prolonged wait. Inconsistent delivery of spectacles and other necessary eye care services is also caused by budgetary restrictions and procurement irregularities, which worsen delays (Masemola et al. 2025). Consequently, patients relied on alternative but less effective vision correction devices such as spectacles while waiting for contact lens fitting appointment (Nkoana, Moodley & Mashige, 2023).

### Outcomes and impact of extended lead time

The protracted lead times associated with optometry services can significantly impact patient flow and healthcare system capacity, creating a ripple effect that extends far beyond the immediate inconvenience to patients. Extended WTs can overwhelm primary care facilities as individuals with unmet eye care needs seek attention, further straining the system’s ability to provide timely and effective care. This bottleneck at the primary care level can result in increased pressure on emergency departments. Patients experiencing acute eye problems may resort to seeking care in already overburdened emergency departments because of the inability to secure timely appointments with optometrists or ophthalmologists (Egbujie et al. 2018). Similar results were found in research carried out in the province of Limpopo, where referral delays resulted in significant WTs for patients in need of specialist care, which had a detrimental impact on the patients’ health outcomes (Masemola et al. 2025).

#### Delayed diagnosis and treatment of other conditions

The increased focus on managing acute eye care needs in primary care settings can divert resources and attention away from other chronic conditions, potentially leading to delays in their diagnosis and management. Reduced patient satisfaction from long WTs can impact the patients’ trust in the healthcare system and their adherence to treatment plans (Abdool, Naidoo & Visser 2022). Moreover, the delays in diagnosis and treatment caused by extended lead times can contribute to a backlog of patients requiring specialised care. Long WTs are made worse by this backlog, which feeds a vicious cycle that is hard to escape (Abdool et al. 2022).

#### Increased demand for complex interventions

As eye conditions progress, patients may require more complex and costly interventions, further straining healthcare resources. Reduced capacity for new patients and the backlog of existing patients can limit the healthcare system’s capacity to accommodate new patients, perpetuating the cycle of long wait times (Verwey & Mahomed 2020). Addressing the issue of lengthy lead times in optometry services requires a multifaceted approach that considers not only the immediate needs of patients but also the broader impact on healthcare system capacity and resource allocation. By optimising patient flow, improving efficiency and expanding access to timely eye care, healthcare systems can mitigate the negative consequences of extended wait times and ensure the delivery of high-quality optometry services to all who need them (Egbujie et al. 2018).

### International trends on wait times

#### Patient registration

This is the first point of service in any healthcare service facility where the patient’s previous history of care is sourced, and registration for the current consultation is done. Though there is a gross lack of South African data, international statistics are used as a baseline. As the first point of entry into the healthcare facility, WTs become one of the critical determinants of the perceived quality of the service received. In a US-based study, reported WTs of 42 min–50 min at the registration department, which, upon intervention, were reduced to an average of 6.55 min (Yu & Yang 2008). In another Indian study, the walk-in registration process took an average of 60 min, while patients who self-registered online took an average of 15 min (Nkoana et al. 2023). Human resources and infrastructure challenges have the potential to cause longer WTs in South Africa, especially in rural provinces. A South African Limpopo-based study (Nkoana et al. 2024) reported a lack of an interactive and online medical record-keeping system, where the current online system only generated patients’ reference numbers to use for physical medical records and payments. The system was further vulnerable; in isolated incidents, patient files were missing. In addition, because of budget cuts, there were difficulties in filling posts to curb the needs of the hospital against the patient turnover. The authors found no recorded lead or WTs for the patient registration department in South Africa.

#### Triage

Australia developed the Australian National Triage Scale (NTS) to standardise triage times across emergency departments (EDs) across hospitals, which was adopted and adapted in Canada, the US and the United Kingdom (UK) (Canadian Institute for Health Information 2007; Crosse 2010; FitzGerald et al. 2010; Fry & Burr 2002; Martins, De Castro Dominguez Cuña & Freitas 2009). While this was more critical to the ED, it is today applied across all hospital departments, including the non-emergency and outpatient departments (OPDs), where patients consult for physiotherapy, dietetics and nutrition, speech therapies, eye care and many other similar services (Edwards 2024). Triage for ED depends on the urgency of the condition of the patient but ranges between 0 min or immediate attention for cases requiring resuscitation to 120 min for non-urgent cases in the UK (Canadian Institute for Health Information 2007; Crosse 2010; FitzGerald et al. 2010; Fry & Burr 2002; Martins et al. 2009). The standardised triage time for ED in South Africa developed by the Cape Town Triage System uses similar time guidelines but its effectiveness and applicability are suspect for the rural settings. Further, the researchers could not find any literature on triage time for OPDs in South Africa.

#### Optometry services

A comprehensive patient examination constitutes examination of the eye health using slit lamp biomicroscopy for ocular adnexa and the anterior segment of the eye, ophthalmoscopy for internal observation, including posterior segment evaluation, full refraction, diagnosis and management of the patient by providing patient education, management with medication, spectacles, contact lenses and other optical devices (Nkoana et al. 2024). This can be a tedious process due to challenges of a lack of equipment, resources and the long queues of patients. Optometrists must work faster than normal to assist most, if not all persons consulting on any given day. Hence, observations are that case-based assessments are conducted, and some of the patients may not be referred to an optometrist if they consult at a facility that employ ophthalmic nurses who then manage pathology and only refer for refraction. This is not applied consistently in all hospitals; hence, some take longer than others. First-time examinations take longer than repeat examinations; routine examinations take shorter times than specialised exams such as contact lens fitting or dilation of patients, and more time may be required if patients have underlying systemic conditions.

#### Pharmacy department

The Pharmacy department is a service point where all patients from respective OPDs usually collect their medication and hence may be overwhelmed. Namibian-based (Magesa, Hanyanya & Erraso 2021) and Nigerian-based (Afolabi & Erhun 2005) studies reported 5 min–25 min and 17.09 min, respectively, for pharmacy WTs. Automated queuing technology, telepharmacy and automated pharmacy devices or machines for quick and accurate filling and dispensing have improved flow (Ritshidze.org.za 2023).

### South African legal framework on waiting times

The existing legal framework on lead and WTs in South Africa was prompted and developed to ensure timely equitable access to healthcare. Table 1 was adapted from the framework. As outlined in the framework, patients are expected to spend shorter times, between 60 min and 120 min, in specialised, district and regional hospitals waiting for the service, while the times spent receiving the service are 60 min for specialised and district hospitals. For regional hospitals, though, an additional 60 min is expected, totalling 120 min for time spent receiving service and another 120 min for waiting to receive the service (Department of Health 2023).

**TABLE 1:** Expected waiting times in public facilities.

Level of hospital	Waiting time		Time spent receiving service		Total lead time spent	
	Minutes	Hours	Minutes	Hours	Minutes	Hours
Specialised	60	1	60	1	120	2
District	12	2	60	1	180	3
Regional	120	2	120	2	240	4
Tertiary / Central	140	2½	190	3	320	5½

*Source:* Department of Health, 2023, National guideline on management of patient waiting time in clinics, community health centres and Outpatients Departments of Public Hospitals of South Africa, viewed 18 March 2026, from https://www.health.gov.za/wp-content/uploads/2024/05/Approved-national-guideline-on-management-of-PWT-final.pdf

Irrespective of these guidelines, the challenge of long patient WTs remains prevalent (Department of Health 2023). Ritshidze.org.za (2023) reported that facility wait times have decreased by more than 25% since 2022, averaging 3:07 h instead of 4:22 h. Improvement is still somewhat erratic, though, the average wait time in KwaZulu-Natal, Limpopo and Mpumalanga is less than 3 h, whereas the average wait time in the Western Cape and Free State is more than 4 h (Ritshidze.org.za 2023). Of the 419 evaluated institutions, 32 (8%) had wait times longer than 5 h, and eight had wait times longer than 6 h. Buthelezi and Van Staden (2020) conducted a study in KwaZulu-Natal and found that only 64.3% of the patients could see an optometrist on the same day as ‘walk-in patients’. In some institutions, 25% of patients waited for up to three weeks before being able to consult with an optometrist. During the time motion audit conducted in this study, it was observed that waiting times varied across facilities, and were also influenced by the size and efficiency of the eye care team. When an optometrist commence clinical duties in each day, patients commonly waited between two and four hours before receiving an eye examination. Similar prolonged waiting times in public eye care services have been reported by Buthelezi and Van Staden (2020). From the moment the optometrist began working for the day, people often had to wait between 2 h and 4 h to be evaluated in the eye clinic (Buthelezi & Van Staden 2020).

## Discussion

The review draws parity between the WT as the idle time a patient waits at any service point before they receive care and a lead time as the cumulative service and WT across various service points, including registration, triage, examination and pharmacy process (Ali et al. 2025; Biya 2022; Pozzan et al. 2025). While this review did not fully focus on the service time, it acknowledged that the increased WT was a significant contributor to longer lead times and operational inefficiency. From this review, structural weaknesses such as a shortage of eyecare professionals in the public service have been touted as contributory factors that increase lead and WTs. With high patient loads, WTs become excessive (Hansraj et al. 2024). Besides the staff shortages, other challenges such as lack of equipment, limited workspace and outdated technologies reduce the service capacity, consequently increasing WTs (Masemola et al. 2025; Zulu & Van Staden 2023). The review found that in some instances, ill-defined administrative processes may likely cause delays in activities such as provision of slots for contact lens fitting and the provision of spectacles and other optical devices (Masemola et al. 2025). These challenges have a negative bearing on the patient’s clinical and behavioural outcomes (Masemola et al. 2025; Mbwogge et al. 2022). Patients may experience severe progression of their eye conditions leading to vision loss while waiting for an appointment to see an eye care professional (Nkoana et al. 2023). With these experiences, patients lose their trust in the health care system, and some become despondent about attending the follow-up consultation sessions. Healthcare facilities may apply operations management principles in line with the queuing theory to predict patient demand and adjust staffing levels. Value stream mapping and improved scheduling may assist in limiting non-value-adding steps to reduce delays in the system of care (Lawal et al. 2014).

### Limitations and future research

Although there is a significant amount of data on related areas of optometry services at public hospitals in South Africa, lead times require further targeted research. To the best of the author’s knowledge, there is limited research on this concept, and more research needs to be done on lead time in optometry services at public hospitals. Future researchers need to address some of the gaps identified in this study, such as examining the complete patient journey from registration, triage and supplementary services to consultation, which is essential for locating bottlenecks and putting plans in place to improve patient happiness and service effectiveness.

## Conclusion

Long waiting and lead times in public optometry services are present because of system inefficiencies, including staff shortages, infrastructure limits, referrals and ineffective supply chains. Redesigning the workflow, improving resource allocation, including human and capital infrastructure and optimisation of the process, waiting and lead times can be reduced.

## References

[CIT0001] Abdool, Z., Naidoo, K. & Visser, L., 2022, ‘Development of a diabetic retinopathy screening model for a district health system in Limpopo Province, South Africa’, *African Vision and Eye Health* 81(1), 568. 10.4102/AVEH.V81I1.568

[CIT0002] Afolabi, M.O. & Erhun, W.O., 2005, ‘Patients’ response to waiting time in an out-patient pharmacy in Nigeria’, *Tropical Journal of Pharmaceutical Research* 2(2), 207–214. 10.4314/TJPR.V2I2.14601

[CIT0003] Ali, M.A., Milani, F. & Dumas, M., 2025, ‘Data-driven identification and analysis of waiting times in business processes’, *Business & Information Systems Engineering* 67(2), 191–208. 10.1007/s12599-024-00868-5

[CIT0004] Ameh, N., Sabo, B. & Oyefabi, M.O., 2013, ‘Application of queuing theory to patient satisfaction at a tertiary hospital in Nigeria’, *Nigerian Medical Journal* 54(1), 64–67. 10.4103/0300-1652.108902 23661902 PMC3644748

[CIT0005] Arksey, H. & O’Malley, L., 2005, ‘Scoping studies: towards a methodological framework’, *International Journal of Social Research Methodology* 8, 19–32. 10.1080/1364557032000119616

[CIT0006] Biya, M., Gezahagn, M., Birhanu, B., Yitbarek, K., Getachew, N. & Beyene, W., 2022, ‘Waiting time and its associated factors in patients presenting to outpatient departments at Public Hospitals of Jimma Zone, Southwest Ethiopia’, *BMC Health Services Research* 22(1), 107. 10.1186/s12913-022-07502-8 35078474 PMC8790858

[CIT0007] Blanford, J.I., Kumar, S., Luo, W. & MacEachren, A.M., 2012, ‘It’s a long, long walk: Accessibility to hospitals, maternity and integrated health centres in Niger’, *International Journal of Health Geographics* 11(1), 24. 10.1186/1476-072X-11-24 22737990 PMC3515413

[CIT0008] Brindley, C., Lomas, J. & Siciliani, L., 2023, ‘The effect of hospital spending on waiting times’, *Health Economics* 32(11), 2427–2445. 10.1002/HEC.4735 37424194

[CIT0009] Buthelezi, L.M. & Van Staden, D., 2020, ‘Integrating eye health into policy: Evidence for health systems strengthening in KwaZulu-Natal’, *African Vision and Eye Health* 79(1), 1–10. 10.4102/AVEH.V79I1.549

[CIT0010] Canadian Institute for Health Information, 2007, *Understanding emergency department wait times: How long do people spend in emergency departments in Ontario?* viewed 26 May 2025, from https://publications.gc.ca/collections/collection_2007/cihi-icis/H118-31-3-2007E.pdf.

[CIT0011] Census, 2022, *MEDIA RELEASE: Census 2022 population count results 10 October 2023*, Statistics South Africa, viewed 26 May 2025, from https://www.statssa.gov.za/?p=16716.

[CIT0012] Chen, J., Bentley, S.A., McKendrick, A.M., Thompson, S.C., Turner, A.W. & Alam, K., 2025, ‘Rural eye care access, workforce challenges and opportunities: Perspectives of the eye health workforce in Western Australia’, *Australian Journal of Rural Health* 33(1), e70004. 10.1111/AJR.70004 39887538 PMC11780685

[CIT0013] Crosse, M., 2010, *Hospital emergency departments: Crowding continues to occur, and some patients wait longer than recommended time frames*, Diane Publishing, Washington, DC.

[CIT0014] Department of Health, 2023, *National guideline on management of patient waiting time in clinics, community health centres and Outpatients Departments of Public Hospitals of South Africa*, viewed 18 March 2026, from https://www.health.gov.za/wp-content/uploads/2024/05/Approved-national-guideline-on-management-of-PWT-final.pdf.

[CIT0015] Ditibane, B., 2024, *Patients criticise Mahikeng Provincial Hospital for long delays in optometry services*, viewed 26 May 2025, from https://health-e.org.za/2024/08/26/patients-criticise-mahikeng-provincial-hospital-for-long-delays-in-optometry-services/.

[CIT0016] Edwards, S.E., 2024, ‘A practical approach to outpatient triage’, *Paediatrics and Child Health* 34(6), 197–199. 10.1093/pch/pxab086

[CIT0017] Egbujie, B.A., Grimwood, A., Mothibi-Wabafor, E.C., Fatti, G., Tshabalala, A.M.E.T., Allie, S. et al., 2018, ‘Impact of “Ideal Clinic” implementation on patient waiting time in primary healthcare clinics in KwaZulu-Natal Province, South Africa: A before-and-after evaluation’, *South African Medical Journal* 108(4), 311–318. 10.7196/SAMJ.2017.V108I4.12583 29629682

[CIT0018] FitzGerald, G., Jelinek, G.A., Scott, D. & Gerdtz, M.F., 2010, ‘Emergency department triage revisited’, *Emergency Medical Journal* 27(2), 86–92. 10.1136/EMJ.2009.077081 20156855

[CIT0019] Fry, M. & Burr, G., 2002, ‘Review of the triage literature: Past, present, future?’, *Australian Emergency Nursing Journal* 5(2), 33–38. 10.1016/S1328-2743(02)80018-9

[CIT0020] Hansraj, R., Dlamini, N., Khan, S., Mtolo, P.C., Ntuli, N.G., Prithipal, C. et al., 2024, ‘Ocular therapeutics and the profession of optometry in South Africa’, *African Journal of Primary Health Care & Family Medicine* 16(1), 4140. 10.4102/phcfm.v16i1.4140 38299541 PMC10839208

[CIT0021] Lawal, A.K., Rotter, T., Kinsman, L., Sari, N., Harrison, L., Jeffery, C. et al., 2014, ‘Lean management in health care: Definition, concepts, methodology and effects reported (systematic review protocol)’, *Systematic Review* 3(1), 103. 10.1186/2046-4053-3-103 PMC4171573 25238974

[CIT0022] Magesa, E., Hanyanya, J. & Erraso, W., 2021, ‘Patient’s satisfaction at outpatient pharmacy department in Intermediate Hospital Oshakati, Oshana region, Namibia’, *GSC Biological and Pharmaceutical Sciences* 14(2), 22–28. 10.30574/GSCBPS.2021.14.2.0040

[CIT0023] Martins, H.M.G., De Castro Dominguez Cuña, L.M. & Freitas, P., 2009, ‘Is Manchester (MTS) more than a triage system? A study of its association with mortality and admission to a large Portuguese hospital’, *Emergency Medicine Journal* 26(3), 183–186. 10.1136/emj.2008.060780 19234008

[CIT0024] Masemola, H.C., Baloyi, O. & Xulu-Kasaba, Z.N., 2025, ‘Evaluating eye care services in South Africa’s Limpopo province using Donabedian’s framework: Insights into structures, processes, and outcomes’, *Clinical Optometry* 17, 115–126. 10.2147/OPTO.S509445 40161271 PMC11955184

[CIT0025] Mashige, K.P., Oduntan, O.A. & Hansraj, R., 2015, ‘Opinions of South African optometry students about working in rural areas after graduation’, *African Journal of Primary Health Care and Family Medicine* 7(1), 1–7. 10.4102/phcfm.v7i1.799 PMC4564836 26245620

[CIT0026] Mbwogge, M., Astbury, N., Nkumbe, H.E., Bunce, C. & Bascaran, C., 2022, ‘Waiting time and patient satisfaction in a subspecialty eye hospital using a mobile data collection kit: Pre-post quality improvement intervention’, *Journal of Medical Internet Research* 3(3), e34263. 10.2196/34263 PMC10414230 37725529

[CIT0027] Moher, D., Liberati, A., Tetzlaff, J. & Altman, D.G., 2009, ‘Preferred reporting items for systematic reviews and meta-analyses: the PRISMA statement’, *BMJ* 339, b2535. 10.1136/bmj.b2535 19622551 PMC2714657

[CIT0028] Nkoana, P.M.W., 2024, ‘Optopreneurship for a competitive and sustainable optometry practice in South Africa’, *African Vision and Eye Health* 83(1), a868. 10.4102/AVEH.V83I1.868

[CIT0029] Nkoana, P.M.W., Mashige, K.P. & Moodley, V.R., 2024, ‘Strengthening keratoconus management systems in South African public sector facilities’, *African Vision and Eye Health* 83(1), 1–11. 10.4102/AVEH.V83I1.832

[CIT0030] Nkoana, P.M.W., Moodley, V.R. & Mashige, K.P., 2023, ‘Keratoconic patient profile and management at public sector facilities in South Africa’, *African Vision and Eye Health* 82(1), a780. 10.4102/AVEH.V82I1.780

[CIT0031] Palvannan, R.K. & Teow, K.L., 2012, ‘Queueing for healthcare’, *Journal of Medical Systems* 36(2), 541–547. 10.1007/S10916-010-9499-7 20703697

[CIT0032] Peta, D., Day, A., Lugari, W.S., Gorman, V., Ahayalimudin, N.A. & Pajo, V.M.T., 2023, ‘Triage: A global perspective’, *Journal of Emergency Nursing* 49, 814–825. 10.1016/J.JEN.2023.08.004 37925222

[CIT0033] Peter, P.O. & Sivasamy, R., 2021, ‘Queueing theory techniques and its real applications to health care systems – Outpatient visits’, *International Journal of Healthcare Management* 14, 114–122. 10.1080/20479700.2019.1616890

[CIT0034] Pozzan, C., Tiso, A., Pamich, C. & Verbano, C., 2025, ‘Sustainable care quality improvement: A scoping literature review of performance measurement in lean healthcare implementations’, *BMC Health Services Research* 25(1), 1452. 10.1186/S12913-025-13598-5 41204235 PMC12595819

[CIT0035] Ritshidze.org.za, 2023, *Ritshidze survey of over 22 000 patients in over 400 clinics across South Africa reveals progress and persistent challenges for public health users*, viewed 12 May 2025, from https://ritshidze.org.za/ritshidze-survey-of-over-22000-patients-in-over-400-clinics-across-south-africa-reveals-progress-and-persistent-challenges-for-public-health-users/.

[CIT0036] Robinson, F.G., Cunningham, L.L., Turner, S.P., Lindroth, J., Ray, D., Khan, T. et al., 2016, ‘Improving a dental school’s clinic operations using lean process improvement’, *Journal of Dental Education* 80, 1170–1179. 10.1002/J.0022-0337.2016.80.10.TB06199.X 27694290

[CIT0037] Solomon, S.D., Shoge, R.Y., Ervin, A.M., Contreras, M., Harewood, J., Aguwa, U.T. et al., 2022, ‘Improving access to eye care: A systematic review of the literature’, *Ophthalmology* 129, e114–e126. 10.1016/J.OPHTHA.2022.07.012 36058739

[CIT0038] Timothy, C.G., Van Staden, D.W., Chepkeitany, H.C., Osuagwu, L.U. & Shaviya, N., 2023, ‘Knowledge, attitude, perception and education on contact lenses for refractive errors in Kenya’, *African Vision and Eye Health* 82(1), 1–8. 10.4102/AVEH.V82I1.738

[CIT0039] Verwey, V.F. & Mahomed, S., 2020, ‘Burden of eye conditions at a specialised eye hospital in KwaZulu-Natal, South Africa’, *African Vision and Eye Health* 79(1), 1–5. 10.4102/AVEH.V79I1.518

[CIT0040] Woldeyohanins, A.E., Molla, N.M., Mekonen, A.W. & Wondimu, A., 2025, ‘The availability and functionality of medical equipment and the barriers to their use at comprehensive specialized hospitals in the Amhara region, Ethiopia’, *Frontiers in Health Services* 4, 1470234. 10.3389/frhs.2024.1470234 39839252 PMC11748297

[CIT0041] Yu, Q. & Yang, K., 2008, ‘Hospital registration waiting time reduction through process redesign’, *International Journal of Six Sigma and Competitive Advantage* 4(3), 240–253.

[CIT0042] Zulu, N.L. & Van Staden, D., 2023, ‘Experiences and perceptions of undergraduate optometry students towards public eye care services in South Africa’, *African Vision and Eye Health* 82(1), 1–7. 10.4102/AVEH.V82I1.726

